# Complete Genome Sequences of Two Strains of Francisella tularensis subsp. *holarctica* bv. japonica

**DOI:** 10.1128/MRA.01127-20

**Published:** 2020-11-05

**Authors:** Akitoyo Hotta, Osamu Fujita, Kiyoshi Tanabayashi, Akihiko Uda, Junji Shindo, Chun-Ho Park, Hisaaki Sato, Michio Suzuki, Shigeru Morikawa, Ken Maeda

**Affiliations:** a Department of Veterinary Science, National Institute of Infectious Diseases, Tokyo, Japan; b Division of Biosafety Control and Research, National Institute of Infectious Diseases, Tokyo, Japan; c Department of Wildlife Science, School of Veterinary Medicine, Kitasato University, Aomori, Japan; d Department of Veterinary Pathology, School of Veterinary Medicine, Kitasato University, Aomori, Japan; e Department of Microbiology, School of Veterinary Medicine, Kitasato University, Aomori, Japan; f Department of Microbiology, Faculty of Veterinary Medicine, Okayama University of Science, Ehime, Japan; Broad Institute

## Abstract

Francisella tularensis, a highly infectious bacterium, is the etiological agent of the zoonotic disease tularemia. It is widely distributed in the Northern Hemisphere, including Japan. Here, we have determined the complete genome sequences of two strains of F. tularensis subsp. *holarctica* bv. japonica isolated from hares in 2008 and 2009.

## Abstract (precis)

Francisella tularensis, a highly infectious bacterium, is an etiological agent of the zoonotic disease tularemia. It is widely distributed in the Northern Hemisphere, including Japan. Here, we have determined the complete genome sequences of two strains of F. tularensis subsp. *holarctica* bv. japonica isolated from hares in 2008 and 2009.

## ANNOUNCEMENT

Francisella tularensis, a Gram-negative coccobacillus, is the etiological agent of the zoonotic disease tularemia. Tularemia naturally occurs in lagomorphs and rodents and has reportedly infected various species of mammals (1). Two subspecies, F. tularensis subsp. *tularensis* and F. tularensis subsp. *holarctica*, are pathogenic to humans. F. tularensis subsp. *tularensis* can be classified into two major clades, A1 and A2 (2). F. tularensis subsp. *holarctica* strains are classified into biovars I, II, and japonica according to their biochemical and geographical data (1) and are genetically classified into 4 clades (2). F. tularensis subsp. *holarctica* bv. japonica is the most basally branching clade of F. tularensis subsp. *holarctica* and is involved in the genetic lineage B.16. This genetic lineage includes F. tularensis strains isolated from Australia, Tibet, and Turkey (2). Complete genome sequence data of Tibetan and Turkish isolates have been published; however, no complete genome of Japanese F. tularensis has been reported.

Here, we present complete genome sequences of F. tularensis subsp. *holarctica* bv. japonica strains NVF1 and KU-1, isolated from carcasses of Japanese hare in 2009 (3) and 2008 (4), respectively. Genomic DNA was extracted from each strain cultured on Eugon chocolate agar at 37°C for 2 days without air regulation (5) using an illustra bacterial genomicPrep mini spin kit (GE Healthcare, Buckinghamshire, UK). All procedures were performed in the biosafety laboratory at the National Institute of Infectious Diseases. Whole-genome *de novo* assembly sequencing was performed by Macrogen Corp. Japan (Kyoto, Japan) using a 20-kb SMRTbell library (PacBio DNA/polymerase binding kit P6) on the RS II sequencing platform. The PacBio DNA sequencing kit 4.0 and 8 single-molecule real-time (SMRT) cells were used for sequencing. The total numbers of reads obtained from NVF1 and KU-1 were 146,146 and 150,504 with *N*_50_ lengths of 15,183 and 14,619 bp, respectively. *De novo* assembly was performed using the hierarchical genome assembly process 3 method (https://github.com/ben-lerch/HGAP-3.0). As a result, single circular contigs were assembled from both NVF1 and KU-1. The completeness of the final assemblies was assessed using the Benchmarking Universal Single-Copy Orthologs (V.3.0) software (https://busco.ezlab.org) (6) with the bacterial database odb9, and scores produced for NVF1 and KU-1 were 93.24% and 93.92% (148 complete sets), respectively. The lengths of the NVF1 and KU-1 genomes were 1,907,706 and 1,907,828 bp, respectively, with a GC content of 32.18% for both (Table 1). Genome sequences were annotated using the DDBJ Fast Annotation and Submission Tool (DFAST; https://dfast.nig.ac.jp) (7).

**TABLE 1:** Francisella tularensis strains analyzed in this study

Strain	Accession no.		Total subread bases	*N*_50_ (bp)	Size (bp)	GC content(%)	Host	Geographical origin of data	
	GenBank	SRA						Latitude (north)	Longitude (east)
NVF1	AP023459	DRR240453	1,571,945,251	15,183	1,907,706	32.18	Japanese hare	39.28	139.93
KU-1	AP023460	DRR240454	1,561,547,887	14,619	1,907,828	32.18	Japanese hare	40.97	141.25

### Data availability.

These sequences have been deposited in the DDBJ under the BioProject number PRJDB10003, and the accession numbers of genomes for NVF1 and KU-1 are AP023459 and AP023460, respectively. The sequences have been submitted to the Sequence Read Archive under the accession numbers DRR240453 and DRR240454, respectively.
